# Organic acid-associated antimicrobial and antibiofilm activity of *Lactobacillus rhamnosus* ATCC 7469 against *Acinetobacter baumannii* and *Staphylococcus aureus*

**DOI:** 10.1007/s42770-026-02095-4

**Published:** 2026-09-24

**Authors:** Mariana Silveira Mello Silva, Priscila Cristina Bartolomeu Halicki, Carlos James Scaini, Jean Lucas de Oliveira Arias, Daniela Fernandes Ramos, Luciana Farias da Costa Avila

**Affiliations:** 1 Parasitology Laboratory, Federal University of Rio Grande (FURG). Faculty of Medicine (FAMED), Federal University of Rio Grande (FURG), General Canabarro, 102, Rio Grande, RS 96200-200 Brazil; 2 New drug development Laboratory (LADEFA), FURG. Faculty of Medicine (FAMED), Federal University of Rio Grande (FURG), General Canabarro, 102, Rio Grande, RS 96200-200 Brazil; 3 Burnett School of Biomedical Sciences, University of Central Florida (UCF), 6900 Lake Nona Blvd, Orlando, FL 32827 USA; 4 Integrated Analysis Center, School of Chemistry and Food, Federal University of Rio Grande, Av. Italia, Km 6 - Campus Carreiros, Rio Grande, Rio Grande do Sul CEP 96203- 900 Brazil

**Keywords:** Biofilms, Probiotics, Drug resistance, *Lactobacillus rhamnosus*, Antimicrobial resistance

## Abstract

Considering the relevance of biofilms in bacterial resistance and the need for innovative therapies against pathogenic microorganisms, alternative strategies using probiotics as antimicrobial agents have emerged. This study aimed to evaluate the antimicrobial and antibiofilm activities of the cell-free supernatant (CFS) of *Lactobacillus rhamnosus* ATCC 7469 against biofilm-forming strains of *Acinetobacter baumannii* and *Staphylococcus aureus*. The activity of CFS was evaluated against *A. baumannii* ATCC 19606, *A. baumannii* 301, *S. aureus* ATCC 12598, and *S. aureus* ATCC 29213. Antimicrobial activity against planktonic cells, inhibition of biofilm formation, and destruction of mature biofilms were assessed at different CFS dilutions. The corresponding concentrations of lactic and acetic acids were determined by high-performance liquid chromatography (HPLC). CFS showed high antimicrobial activity, with growth inhibition ranging from 88.26% to 100% at the two highest concentrations tested (197.8/108.7 and 107.0/54.5 mM lactic/acetic acid). At 57.8/28.3 mM, growth inhibition ranged from 60.05% to 81.65%, whereas at 27.2/13.5 mM it ranged from 21.12% to 41.03%. CFS also strongly inhibited biofilm formation, with inhibition rates ranging from 66.17% to 100%, whereas destruction of mature biofilms ranged from 24.75% to 88.11%. HPLC analysis demonstrated the presence of lactic and acetic acids in the CFS, with lactic acid being the predominant organic acid detected. Neutralization of the CFS to pH 7.0 markedly reduced its antimicrobial and antibiofilm activities, supporting an important contribution of acidity and acid-associated components to the observed effects. Overall, *L. rhamnosus* ATCC 7469 CFS exhibited substantial antimicrobial and antibiofilm activity against *A. baumannii* and *S. aureus*, supporting further investigation of *Lactobacillus*-derived CFS as a potential local antimicrobial strategy.

## Introduction

Biofilms are microbial communities characterized by the production of extracellular polymeric substances that favor adhesion to biotic and abiotic surfaces [1]. It is known that intra and interspecies interactions between bacteria and microorganisms affect the development of biofilms. Bacteria in biofilms differ metabolically from their planktonic counterparts in pure culture, as they are more resistant to antibiotics and disinfectants [2]. In 2017, the World Health Organization highlighted noteworthy bacteria with broad resistance to antibiotics, emphasizing that these should be given priority in the investigation of new drugs. In this list, ESKAPE pathogens are classified as critical priority (*Acinetobacter baumannii*,* Pseudomonas aeruginosa*,* Klebsiella pneumoniae*, and *Enterobacter* spp.) and high priority (*Enterococcus faecium* and *Staphylococcus aureus*) for research and discoveries that minimize morbidity and mortality from these infectious agents [3].

Probiotics can be effective in combating pathogenic biofilms by modifying the surrounding environment [4–7]. A recent systematic review revealed that the most promising probiotic strains against different groups of pathogens were *Lactobacillus acidophilus*, *L. casei*, and *L. rhamnosus* for gram-negative bacteria; *L. fermentum* and *L. paracasei* for gram-positive bacteria; and *L. casei* and *L. rhamnosus* for yeasts [8]. The mechanisms used by *Lactobacillus* spp. to combat biofilms are diverse, with the main antimicrobial agents being bacteriocins, biosurfactants, hydrogen peroxide, and lactic acid [7, 9, 10].

Considering the need for innovative therapies to combat resistance acquired by pathogenic microorganisms, a new strategy proposes the use of probiotics as antimicrobial agents. They offer a natural alternative and have demonstrated important health-promoting properties. Additionally, they are generally considered safe for humans and the environment [8]. Thus, this study aimed to evaluate the antimicrobial and antibiofilm potential of the cell-free supernatant (CFS) of *Lactobacillus rhamnosus* ATCC 7469 against bacterial pathogens of clinical interest, thereby contributing to the development of new therapeutic alternatives.

## Materials and methods

### Cultivation of L. rhamnosus ATCC 7469 and CFS isolation

The probiotic *L. rhamnosus* ATCC 7469, obtained from Fundação Oswaldo Cruz (FIOCRUZ, Brazil), was identified by matrix-assisted laser desorption ionization–time of flight mass spectrometry (MALDI-TOF MS) prior to the experiments. The strain was cultivated in 250 mL of Man, Rogosa and Sharpe (MRS) medium and incubated for 24 h in a Tecnal 420 incubator with orbital agitation at 37 °C. The culture was then centrifuged at 4000 × g for 15 min. Afterwards, the supernatant was separated and filtered through a 0.22 μm membrane (Millipore^®^, Darmstadt, Germany) to obtain the cell-free supernatant (CFS). For the neutralized CFS condition, aliquots of CFS were adjusted to pH 7.0 using NaOH prior to the antimicrobial and antibiofilm assays. After preparation, the CFS was diluted in phosphate-buffered saline (PBS) at ratios ranging from 1:2 to 1:16. The neutralized CFS was evaluated in parallel with the non-neutralized CFS at the corresponding concentrations. The CFS preparations were subsequently used in the antimicrobial and antibiofilm assays.

### Biofilm-producing bacteria

The bacteria used in the study were *A. baumannii* ATCC 19606, *A. baumannii* 301, *S. aureus* ATCC 12598, and *S. aureus* ATCC 29213, from the Strain Bank of the New drug development Laboratory (LADEFA) of the Faculty of Medicine of the Federal University of Rio Grande, Brazil. The bacteria were spread on Mueller-Hinton (MH) agar plates and incubated at 37 °C for 24 h. Subsequently, the inoculum was prepared for antimicrobial and antibiofilm activity assays by diluting each bacterial suspension (adjusted to the 0.5 McFarland scale) in MH medium, and MH medium enriched with 0.2% glucose, resulting in final concentrations of approximately 5 × 10^5^ CFU/mL and 1 × 10^6^ CFU/mL, respectively according to the methodology of each assay described below.

### Antimicrobial activity assay: determination of the minimum inhibitory concentration (MIC)

In this assay, the activity of CFS against planktonic cells was evaluated to determine the minimum concentration capable of inhibiting the growth of *A. baumannii* and *S. aureus*. The procedure was performed according to the criteria of the Clinical and Laboratory Standards Institute (CLSI). Briefly, the inocula were prepared by diluting each bacterial suspension (adjusted to the 0.5 McFarland scale) in MH medium at a ratio of 1:100, resulting in a final concentration of approximately 5 × 10⁵ CFU/mL in each well. The MIC of CFS was determined in 96-well polystyrene flat-bottom microtiter plates (CRAL^®^, São Paulo, Brazil) using base-2 serial microdilution assays at ratios ranging from 1:2 to 1:16, in a total volume of 100 µL [11]. Pure MH medium and MRS were used as negative controls, whereas MH medium containing the respective bacterial inoculum was used as the positive control. CFS without bacterial inoculum was included as a blank to account for background absorbance, with corresponding blanks used for the neutralized CFS condition. Both non-neutralized and neutralized CFS were evaluated in parallel at the corresponding concentrations to assess the contribution of CFS acidity to the observed antimicrobial activity. The plates were incubated at 37 °C for 24 h, and the absorbance was measured at 620 nm using a plate reader (Thermo Plate TP-Reader BioTek ELx800^®^, São Paulo, Brazil).

### Antibiofilm activity assay: inhibition of biofilm formation

In this assay, the strains were exposed to different concentrations of CFS before biofilm formation to determine whether CFS inhibits biofilm formation. From growth in MH medium, a bacterial suspension of each strain enriched with 0.2% glucose was prepared at a final concentration of 1 × 10⁶ CFU/mL [11]. The suspension (100 µL) was transferred to a 96-well polystyrene flat-bottom microplate (CRAL^®^, São Paulo, Brazil), where the cells were exposed to CFS concentrations previously selected in the MIC assay. Both non-neutralized and neutralized CFS were evaluated in parallel at the corresponding concentrations to assess the contribution of CFS acidity to the inhibition of biofilm formation. The plates were incubated for 24 h at 37 °C. The biofilm was then washed three times with 1× PBS (100 µL), and the adhered cells were fixed with 100% methanol (100 µL) and stained with 0.4% crystal violet solution (200 µL). Next, the wells were washed three times with distilled water (200 µL), and the dye was dissolved with 100% ethanol (200 µL). Biofilm formation was quantified by measuring the optical density at 620 nm [12].

### Antibiofilm activity assay: destruction of biofilms

In this assay, bacterial biofilms were first allowed to form, and the adhered cells were subsequently exposed to different concentrations of CFS to evaluate its ability to destroy preformed biofilms. After preparation of the inoculum according to the methodology described in the previous assay, the plates were incubated at 37 °C for 24 h to allow biofilm formation. Both non-neutralized and neutralized CFS were evaluated in parallel at the corresponding concentrations to assess the contribution of CFS acidity to the destruction of mature biofilms. The contents of the wells were then removed, and the wells were washed with 1× PBS to eliminate non-adherent cells. Subsequently, 100 µL of MH medium enriched with 0.2% glucose containing CFS at concentrations of 1:2, 1:4, and 1:8 were added. The plates were incubated for another 24 h at 37 °C. The biofilm washing, fixation, and staining steps were then performed as described above, and biofilm biomass was quantified by measuring the OD at 620 nm.

### Data processing

The experiments were performed in experimental triplicate, and the data were processed according to the mean values and standard deviation. One-way ANOVA was used for statistical analysis, followed by Bonferroni’s multiple comparison test. Statistical significance was set at *p* < 0.05.

## Results

### Antimicrobial activity assay (MIC)

**Fig. 1:**
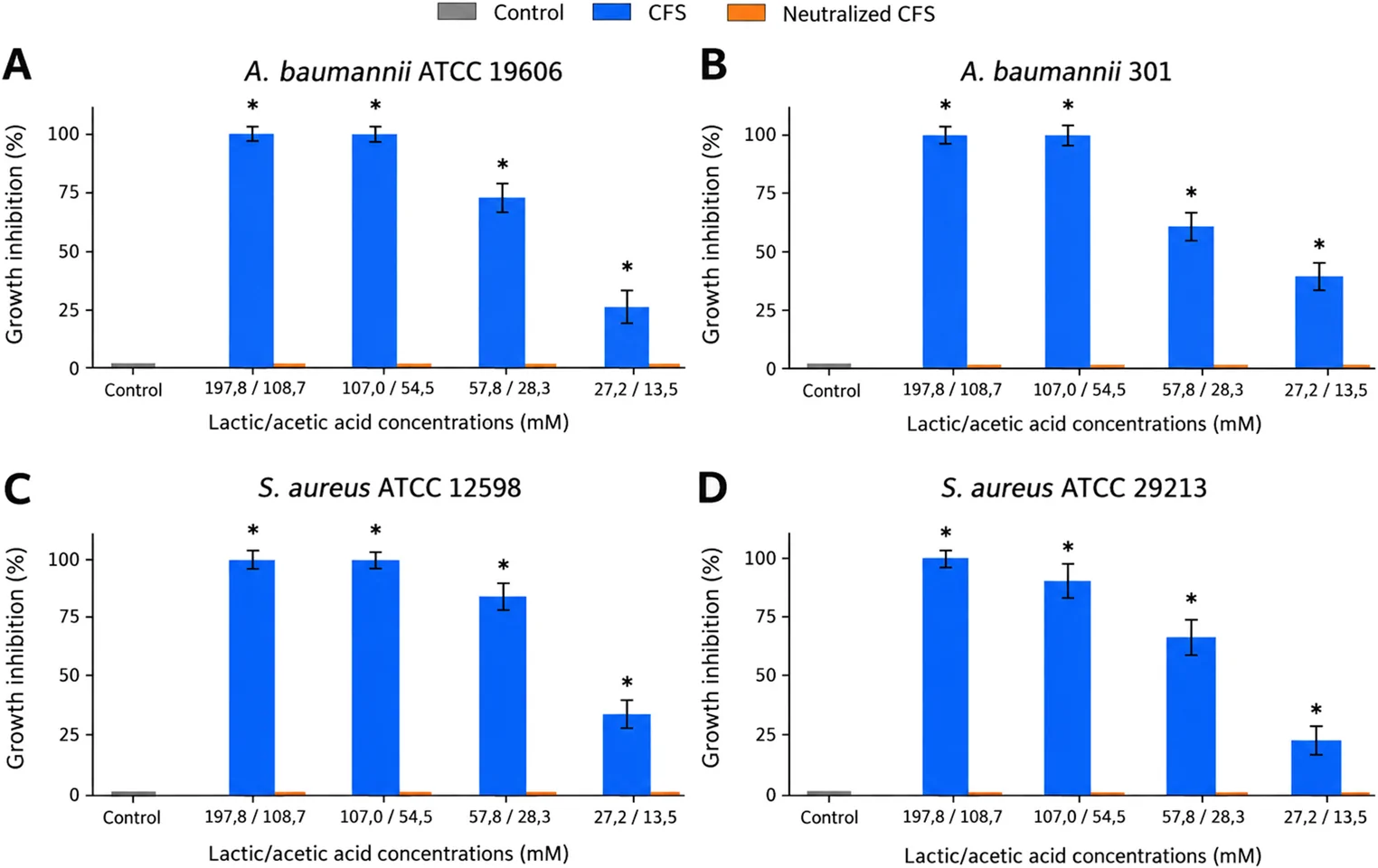
Antimicrobial activity of the cell-free supernatant (CFS) from *Lactobacillus rhamnosus* ATCC 7469 against planktonic cells of *Acinetobacter baumannii* and *Staphylococcus aureus*. Growth inhibition was evaluated at lactic/acetic acid concentrations of 197.8/108.7, 107.0/54.5, 57.8/28.3, and 27.2/13.5 mM, corresponding to CFS dilutions of 1:2, 1:4, 1:8, and 1:16, respectively. Data represent mean growth inhibition (%) ± standard deviation (SD) for *A. baumannii* ATCC 19606 (**A**), *A. baumannii* 301 (**B**), *S. aureus* ATCC 12598 (**C**), and *S. aureus* ATCC 29213 (**D**). CFS and neutralized CFS (pH 7.0) were evaluated in parallel. Values of 0% inhibition were represented as 1% for visualization purposes. Asterisks (*) indicate statistically significant differences between CFS-treated and control groups (*p* < 0.05)

The antimicrobial activity of *L. rhamnosus* ATCC 7469 CFS against planktonic cells was evaluated at lactic/acetic acid concentrations of 197.8/108.7, 107.0/54.5, 57.8/28.3, and 27.2/13.5 mM, corresponding to CFS dilutions of 1:2, 1:4, 1:8, and 1:16, respectively. At 197.8/108.7 mM lactic/acetic acid, growth inhibition ranged from 96.71% for *S. aureus* ATCC 12598 to approximately 100% for *A. baumannii* strains. At 107.0/54.5 mM, growth inhibition ranged from 88.26% for *S. aureus* ATCC 29213 to approximately 100% for *A. baumannii* strains and *S. aureus* ATCC 12598. At 57.8/28.3 mM, growth inhibition ranged from 60.05% for *A. baumannii* 301 to 81.65% for *S. aureus* ATCC 12598. At the lowest concentration tested (27.2/13.5 mM), growth inhibition decreased to 24.28% for *A. baumannii* ATCC 19606, 41.03% for *A. baumannii* 301, 30.85% for *S. aureus* ATCC 12598, and 21.12% for *S. aureus* ATCC 29213 (Fig. 1).

The CFS-treated groups showed statistically significant differences in growth inhibition compared with the respective bacterial controls (*p* < 0.001 for the higher lactic/acetic acid concentrations; *p* < 0.05 for the lowest concentrations). Based on the antimicrobial activity observed, the CFS concentrations corresponding to 197.8/108.7, 107.0/54.5, and 57.8/28.3 mM lactic/acetic acid (1:2, 1:4, and 1:8 dilutions, respectively) were selected for the subsequent antibiofilm assays, whereas the lowest concentration tested (27.2/13.5 mM; 1:16 dilution) was not selected for further analysis.

Neutralization of the CFS to pH 7.0 markedly reduced the antimicrobial activity, with no relevant growth inhibition observed for the neutralized CFS at the concentrations tested.

### Antibiofilm activity assay: inhibition of biofilm formation

Based on the antimicrobial activity results, the CFS concentrations corresponding to 197.8/108.7, 107.0/54.5, and 57.8/28.3 mM lactic/acetic acid (1:2, 1:4, and 1:8 dilutions, respectively) were selected for the subsequent antibiofilm assays. The lowest concentration tested in the antimicrobial assay, corresponding to 27.2/13.5 mM lactic/acetic acid (1:16 dilution), was not included in the antibiofilm assays. All strains evaluated in this study were identified as biofilm-forming strains.

**Fig. 2:**
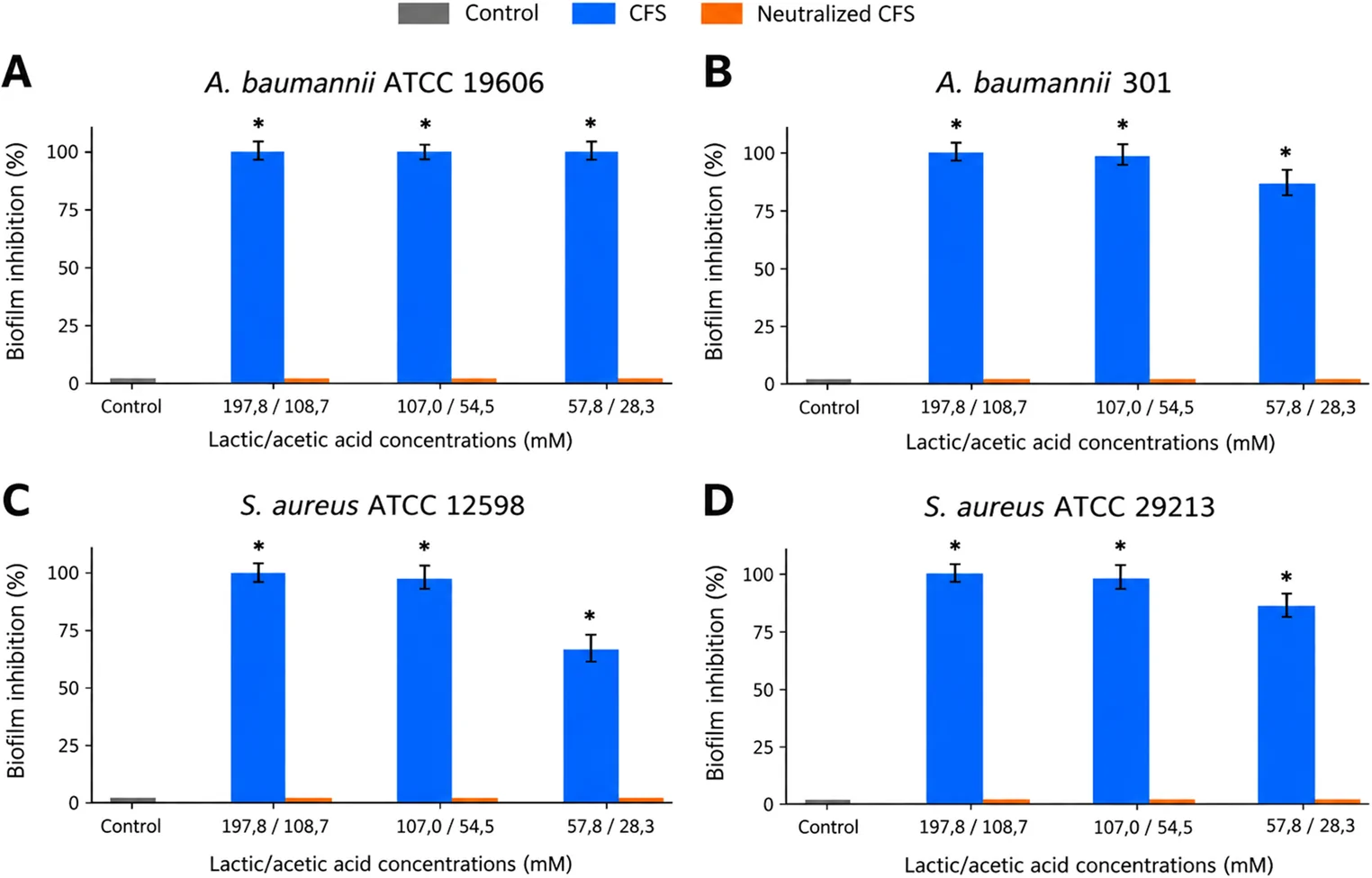
Inhibition of biofilm formation by the cell-free supernatant (CFS) from *Lactobacillus rhamnosus* ATCC 7469 against *Acinetobacter baumannii* and *Staphylococcus aureus*. Biofilm formation inhibition was evaluated at lactic/acetic acid concentrations of 197.8/108.7, 107.0/54.5, and 57.8/28.3 mM, corresponding to CFS dilutions of 1:2, 1:4, and 1:8, respectively. Data represent mean biofilm formation inhibition (%) ± standard deviation (SD) for *A. baumannii* ATCC 19606 (**A**), *A. baumannii* 301 (**B**), *S. aureus* ATCC 12598 (**C**), and *S. aureus* ATCC 29213 (**D**). CFS and neutralized CFS (pH 7.0) were evaluated in parallel. Values of 0% biofilm formation inhibition were represented as 1% for visualization purposes. Asterisks (*) indicate statistically significant differences between CFS-treated and control groups (*p* < 0.05)

The CFS showed high levels of biofilm formation inhibition at the tested lactic/acetic acid concentrations. For *A. baumannii* ATCC 19606, biofilm formation inhibition was 99.37%, 98.69%, and 98.79% at 197.8/108.7, 107.0/54.5, and 57.8/28.3 mM lactic/acetic acid, respectively. For *A. baumannii* 301, the corresponding inhibition values were 99.24%, 98.30%, and 84.82%. For *S. aureus* ATCC 12598, inhibition values were 100%, 93.61%, and 66.17%, whereas *S. aureus* ATCC 29213 showed 95.07%, 94.57%, and 83.73% inhibition at the same concentrations, respectively (Fig. 2).

Statistically significant differences in biofilm formation inhibition were observed between the CFS-treated groups and the respective controls at the tested concentrations (*p* < 0.001). Neutralization of the CFS to pH 7.0 markedly reduced biofilm formation inhibition, with no relevant inhibitory effect observed for the neutralized CFS at the concentrations tested.

### Antibiofilm activity assay: destruction of biofilms

**Fig. 3:**
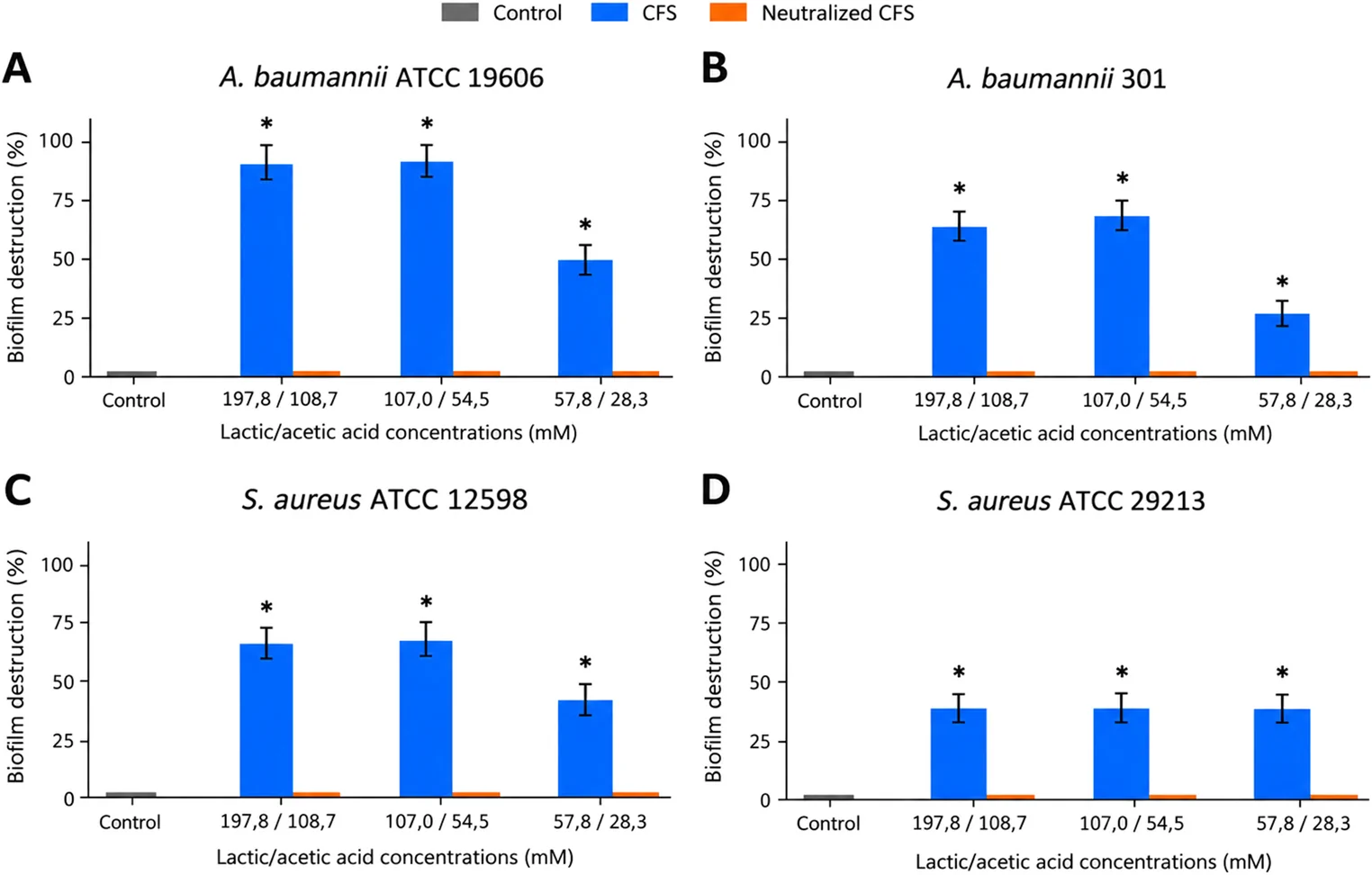
Destruction of mature biofilms by the cell-free supernatant (CFS) from *Lactobacillus rhamnosus* ATCC 7469 against *Acinetobacter baumannii* and *Staphylococcus aureus*. Mature biofilm destruction was evaluated at lactic/acetic acid concentrations of 197.8/108.7, 107.0/54.5, and 57.8/28.3 mM, corresponding to CFS dilutions of 1:2, 1:4, and 1:8, respectively. Data represent mean biofilm destruction (%) ± standard deviation (SD) for *A. baumannii* ATCC 19606 (**A**), *A. baumannii* 301 (**B**), *S. aureus* ATCC 12598 (**C**), and *S. aureus* ATCC 29213 (**D**). CFS and neutralized CFS (pH 7.0) were evaluated in parallel. Values of 0% biofilm destruction were represented as 1% for visualization purposes. Asterisks (*) indicate statistically significant differences between CFS-treated and control groups (*p* < 0.05)

The ability of *L. rhamnosus* ATCC 7469 CFS to destroy preformed biofilms was evaluated at lactic/acetic acid concentrations of 197.8/108.7, 107.0/54.5, and 57.8/28.3 mM, corresponding to CFS dilutions of 1:2, 1:4, and 1:8, respectively. Biofilm destruction ranged from 24.75% to 88.11%, with the greatest effects generally observed at the higher lactic/acetic acid concentrations. Statistically significant differences in biofilm destruction were observed between the CFS-treated groups and the respective controls at the tested concentrations, as indicated in Fig. 3. Neutralization of the CFS to pH 7.0 markedly reduced the biofilm destruction effect, with no relevant destruction observed for the neutralized CFS at the concentrations tested.

###  pH values of CFS concentrations

The pH of the CFS increased progressively with increasing dilution. The measured pH values were 3.6, 4.1, 5.3, and 6.0 for the CFS dilutions of 1:2, 1:4, 1:8, and 1:16, respectively.

### Organic acid profile of the cell-free supernatant

High-performance liquid chromatography (HPLC) analysis demonstrated the presence of lactic and acetic acids in the CFS of *L. rhamnosus* ATCC 7469, with lactic acid being the predominant organic acid detected (Table 1). The concentrations of both organic acids decreased progressively with increasing CFS dilution. Lactic acid concentrations were 17.82, 9.64, 5.21, and 2.45 g/L at CFS dilutions of 1:2, 1:4, 1:8, and 1:16, respectively, whereas acetic acid concentrations were 6.53, 3.27, 1.70, and 0.81 g/L, respectively. The decrease in organic acid concentrations occurred concomitantly with the reduction in antimicrobial and antibiofilm activity observed at the more diluted CFS concentrations (Figs. 1, 2 and 3).

**Table 1:** Organic acid concentrations detected in the cell-free supernatant of *Lactobacillus rhamnosus* ATCC 7469 by high-performance liquid chromatography

CFS dilution	Lactic acid (g/L)	Lactic acid (mM)	Acetic acid (g/L)	Acetic acid (mM)
1:2	17.82	197.8	6.53	108.7
1:4	9.64	107.0	3.27	54.5
1:8	5.21	57.8	1.70	28.3
1:16	2.45	27.2	0.81	13.5

Organic acid concentrations were determined by high-performance liquid chromatography (HPLC) using UV detection at 210 nm

## Discussion

Probiotics are a natural, viable, affordable, and non-toxic treatment alternative, with recognized properties of stimulating host immunity and promoting health [8, 13, 14]. Currently, with the increasing occurrence of multidrug-resistant bacteria and the concern to develop therapeutic strategies that minimize environmental contamination, the use of probiotics as antibacterial agents has revealed promising results [15, 16]. In the present study, an important effect of CFS on bacterial activity and biofilm formation was observed for all strains tested. In this regard, probiotic therapy has been reported to have inhibitory effects against both Gram-positive and Gram-negative bacteria [17]. Previous studies have demonstrated that cell-free supernatants (CFSs) from *Lactobacillus* strains can exert antimicrobial and antibiofilm effects against clinically relevant pathogens. In particular, CFS from *L. rhamnosus* ATCC 7469 has been shown to inhibit *S. aureus* growth and biofilm-associated properties, including surface hydrophobicity, initial adhesion, and biofilm formation [18]. Similarly, *L. rhamnosus* has demonstrated significant antibiofilm activity against *A. baumannii*, with lactic and acetic acids identified as important antibiofilm components [19]. These findings are consistent with the antimicrobial and antibiofilm effects observed for *L. rhamnosus* ATCC 7469 CFS in the present study.

The production of acids by *Lactobacillus* strains is recognized as an important factor in combating pathogens, as it alters the surrounding environment through the release of growth-inhibiting compounds such as organic acids, including lactic, acetic, propionic, and succinic acids [4–7]. In the present study, HPLC analysis confirmed the presence of lactic and acetic acids in the CFS of *L. rhamnosus* ATCC 7469. Importantly, the reduction in organic acid concentrations across serial dilutions occurred concomitantly with the decrease in antimicrobial and antibiofilm activity observed in the biological assays. The highest concentrations of organic acids were detected in the least diluted CFS samples, which also showed the strongest inhibitory effects against planktonic growth and biofilm formation. These findings support a relevant contribution of organic acids to the antimicrobial and antibiofilm activity of the probiotic CFS.

Lactic acid, the predominant organic acid identified in the present study, is known to exert antimicrobial effects through mechanisms that include membrane permeation in its undissociated form, intracellular acidification, disruption of metabolic pathways, ATP depletion, and membrane destabilization [20–23]. Acetic acid may also contribute to increased membrane permeability and impairment of bacterial energy metabolism, negatively affecting both planktonic cells and biofilm establishment [21, 22, 24–26]. The concurrent presence of lactic and acetic acids in the CFS may therefore contribute to the antimicrobial and antibiofilm effects observed against *A. baumannii* and *S. aureus.*

An association was also observed between CFS pH and antimicrobial and antibiofilm activity. The pH increased progressively with CFS dilution, from 3.6 at 1:2 to 6.0 at 1:16, concomitantly with a reduction in antimicrobial and antibiofilm activity. This finding suggests that the physicochemical environment generated by the CFS may contribute to its biological effects. However, the relationship between pH and activity should not be interpreted as a direct effect of pH alone, since organic acid activity is also influenced by the degree of dissociation of these compounds [23]. Thus, the decrease in activity observed with increasing pH may reflect changes in both environmental acidity and the chemical state of the organic acids present in the CFS.

The neutralization experiment provided additional evidence for the involvement of acidity in the observed activity. Neutralization of the CFS to pH 7.0 markedly reduced antimicrobial activity and biofilm-related effects under the conditions tested. This finding is consistent with previous observations using the same probiotic strain. In a study evaluating the CFS of *L. rhamnosus* ATCC 7469 against *S. aureus* ATCC 33591, antimicrobial activity was observed for the non-neutralized CFS, whereas neutralization completely abolished the formation of inhibition zones. The authors also reported that heating the CFS to 100 °C did not affect its antimicrobial activity and suggested that the observed effect was more likely associated with organic acid production than with bacteriocin activity [18]. Similarly, the marked reduction in activity following neutralization in the present study supports an important contribution of acidity and acid-associated components.

The differences in bacterial envelope structure may influence the susceptibility of Gram-positive and Gram-negative bacteria to organic acids. *S. aureus*, a Gram-positive bacterium, possesses a thick peptidoglycan layer but lacks the outer membrane characteristic of Gram-negative bacteria. In contrast, *A. baumannii* has a thinner peptidoglycan layer surrounded by an outer membrane that represents an additional permeability barrier [27, 28]. These structural differences may affect the interaction of lactic and acetic acids with the bacterial envelope and consequently their antimicrobial effects. Organic acids can exert antimicrobial activity not only through environmental acidification but also through their undissociated forms, which can cross bacterial membranes and subsequently dissociate intracellularly, resulting in cytoplasmic acidification and disruption of cellular metabolism [23]. However, the present results did not demonstrate a uniform difference in susceptibility between *A. baumannii* and *S. aureus*. Rather, the response varied among strains and according to the biological endpoint evaluated, indicating that cell envelope structure alone cannot explain the observed activity. Strain-specific acid resistance mechanisms, membrane composition, metabolic state, and biofilm-associated characteristics may also contribute to the differences observed.

The antibiofilm effects of *Lactobacillus*-derived CFS may involve mechanisms beyond direct bacterial growth inhibition. Previous studies have suggested that *Lactobacillus* CFS can interfere with biofilm development through effects on surface adhesion, extracellular polymeric substances, quorum sensing, and virulence-associated pathways [29–32]. In the present study, the high inhibition of biofilm formation observed for both *A. baumannii* and *S. aureus* suggests that the CFS may interfere with early stages of biofilm development. The stronger and more consistent inhibition of biofilm formation compared with the destruction of mature biofilms further suggests that the CFS may be particularly effective at preventing biofilm establishment rather than completely eliminating an already established biofilm.

The activity against preformed biofilms was lower and more variable than that observed for biofilm formation, particularly among the *S. aureus* strains. This difference may reflect the greater structural complexity and physiological heterogeneity of mature biofilms, in which extracellular polymeric substances and altered metabolic states can reduce the accessibility and susceptibility of embedded cells to antimicrobial compounds [33]. From a translational perspective, clinical use would require additional pharmacokinetic and safety evaluation before its feasibility could be assessed. Therefore, further studies using purified organic acids or standardized CFS preparations, appropriate safety assessments, and in vivo models are required before the therapeutic applicability of acidic *Lactobacillus*-derived formulations can be considered [34, 35].

Overall, the results indicate that the CFS of *L. rhamnosus* ATCC 7469 exhibits substantial antimicrobial and antibiofilm activity against *A. baumannii* and *S. aureus*, with lactic and acetic acids identified as important organic components. The marked reduction in activity following neutralization supports an important role of acidity and acid-associated components in the observed effects, while the concomitant decrease in organic acid concentrations and biological activity further supports their contribution. However, the individual effects of lactic acid, acetic acid, and other CFS metabolites remain to be established. Future studies should therefore evaluate these components individually and in combination under controlled pH conditions, as well as investigate the safety, stability, and feasibility of topical formulations.

## Data Availability

The data underlying this article are available in the article and in its online supplementary material.

## Abbreviations

µm: Micrometer
CFS: Cell-free supernatant
CLSI: Clinical and Laboratory Standards Institute
ESKAPE: *Enterococcus faecium, Staphylococcus aureus, Klebsiella pneumoniae,* * Acinetobacter baumannii, Pseudomonas aeruginosa, Enterobacter species*
*L. acidophilus*: *Lactobacillus acidophilus*
*L. casei*: *Lactobacillus casei*
*L. delbrueckii*: *Lactobacillus delbrueckii*
*L. fermentum*: *Lactobacillus fermentum*
*L. helveticus*: *Lactobacillus helveticus*
*L. paracasei*: *Lactobacillus paracasei*
*L. rhamnosus*: *Lactobacillus rhamnosus*
LADEFA: New drug development Laboratory
MH: Mueller-Hinton
MIC: minimum inhibitory concentration
PBS: phosphate-buffered saline
